# Comparative outcomes of microsurgical and endovascular treatment for ruptured and unruptured anterior communicating artery aneurysms

**DOI:** 10.1007/s10143-025-04035-6

**Published:** 2026-01-14

**Authors:** Bekir Can Kendirlioglu, Umid Sulaimanov, Ufuk Erginoglu, Selin Bozdag, Franco Patricio Vera Figueroa, Umut Tan Sevgi, Burcu Kok Kendirlioglu, Cagdas Ataoglu, Abdullah Keles, Abdurrahman Aycan, Miner Ross, Laura Stone McGuire, Azam Syed Ahmed, Mustafa K. Baskaya

**Affiliations:** 1 Department of Neurological Surgery, School of Medicine and Public Health, University of Wisconsin-Madison, CSC K4/822, 600 Highland Avenue, Madison, WI 53792 USA; 2 Department of Psychiatry & Psychology, Mayo Clinic, Rochester, MN 55905 USA

**Keywords:** Aneurysm occlusion, Anterior communicating artery, Endovascular coiling, Microsurgical clipping, Subarachnoid hemorrhage

## Abstract

Anterior communicating artery (AComA) aneurysms remain challenging because of their complex anatomy, rupture risk, and proximity to vital structures. This study compared the long-term durability, retreatment rates, complications, and outcomes of microsurgical clipping and endovascular treatment. This retrospective study included 313 patients (≥ 18 years) with ruptured or unruptured AComA aneurysms treated at a single center (2006–2024). Patients were categorized by treatment modality into two groups: (1) Microsurgical clipping and (2) Endovascular treatment. Primary outcomes included aneurysm occlusion status, retreatment, and functional recovery, measured by the Glasgow Outcome Scale and the modified Rankin Scale. Of the 313 patients, 166 (53.0%) underwent microsurgical clipping, and 147 (47.0%) received endovascular treatment. Complete occlusion was achieved in 98.2% of the microsurgical group compared to 38.1% in the endovascular group (*p* < 0.001). Retreatment was required for 15.6% of endovascular patients (excluding planned two-stage procedures), while no retreatments occurred for the microsurgical group (*p* < 0.001). There were no statistically significant differences in functional outcomes between treatment groups at discharge and follow-up. Mean aneurysm size was smaller for ruptured aneurysms (5.82 mm) than unruptured ones (6.80 mm; *p* = 0.020), and diabetes mellitus was more prevalent in the unruptured group (27.5% vs. 8.1%; *p* < 0.001). Microsurgical clipping provided significantly higher rates of complete occlusion and lower retreatment rates compared to endovascular coiling, with equivalent functional outcomes. These findings support the continued role of microsurgery for treating AComA aneurysms, particularly in patients requiring durable occlusion and less intensive long-term surveillance, although differences in rupture status between the treatment groups should be considered when interpreting these results.

**Supplementary Information:**

The online version contains supplementary material available at 10.1007/s10143-025-04035-6.

## Introduction

Anterior communicating artery (AComA) aneurysms represent one of the most frequent types of intracranial aneurysms, accounting for up to 30–35% of cases [1, 2]. These can be managed through microsurgical clipping (MC) or endovascular treatment (ET), each with distinct advantages and limitations. Due to their deep anatomical location, intimate relationship with vital perforators, and complex hemodynamics, AComA aneurysms present unique therapeutic challenges [3–5].

Although comparative studies exist, choosing between MC and ET is often guided by local expertise, institutional preference, and aneurysm morphology rather than standardized criteria. MC is associated with higher rates of complete occlusion and lower recurrence [6, 7]. In contrast, ET is a less invasive option that has become increasingly preferred in recent years due to lower procedural morbidity. However, ET has a higher rate of incomplete occlusion and aneurysm recanalization over time [8–10]. These challenges are particularly relevant for AComA aneurysms, where complex anatomy can limit treatment options and affect outcomes.

This study aims to compare the long-term outcomes of MC and ET for ruptured and unruptured AComA aneurysms, with a particular focus on complication rates, retreatment frequency, and occlusion durability.

## Methods

### Study design and patient population

This retrospective cohort study included patients with AComA aneurysms treated at a single institution between January 2006 and April 2024. Institutional Review Board (IRB) approval was obtained (IRB #2015 − 0856). Patient records and imaging studies were reviewed to identify eligible cases.

Inclusion criteria were age ≥ 18 years with a radiologically confirmed diagnosis of a ruptured or unruptured AComA aneurysm identified via computed tomography angiography (CTA), magnetic resonance angiography (MRA), or digital subtraction angiography (DSA). In patients with multiple aneurysms, inclusion required confirmation, based on imaging and operative findings, that the ruptured aneurysm was located at the AcomA.

Exclusion criteria comprised non-saccular aneurysms including mycotic or pseudoaneurysms, alternative etiologies of hemorrhage (e.g., arteriovenous malformations or dural arteriovenous fistulas), multiple intracranial hemorrhagic lesions, prior aneurysm treatment performed at another institution, and a less than one-year follow-up duration. Patients younger than 18 years of age were also excluded.

Treatment modality was determined by consensus of a multidisciplinary neurovascular team, considering aneurysm morphology (neck width > 4 mm, dome-to-neck ratio < 1.5, multilobulated/complex projections), subarachnoid hemorrhage (SAH) grade, hydrocephalus, patient age/comorbidities, and the relationship of the aneurysm neck to the dominant A1 segment and adjacent vessels. Microsurgical clipping was generally favored for wide-necks or aneurysms with complex projections, or the patient preferred microsurgery when durable occlusion and perforator preservation were anticipated with open surgery. Whereas, endovascular therapy was preferred for patients with significant comorbidities or advanced age, narrow-neck aneurysms with favorable access, or when surgical exposure was expected to carry high morbidity or the patient preferred endovascular treatment. In poor-grade SAH patients, treatment was individualized, with decisions guided mainly by the presence of hematoma, mass effect, and overall clinical condition rather than a fixed preference for either modality.

A total of 313 patients met the inclusion criteria. For analytical purposes, patients were stratified by treatment modality (MC vs. ET) and aneurysm status (ruptured vs. unruptured). Data were collected from admission to hospital discharge, death, or the most recent clinical follow-up. Baseline characteristics included age, sex, smoking status, and relevant medical history. Aneurysm morphology was recorded, including maximum aneurysm size and dome projection, with the latter assigned according to the Yasargil classification system [11]. In cases of rupture, clinical and radiographic severity were scored according to Hunt-Hess and modified Fisher scales, respectively.

For patients with ruptured aneurysms, cerebral vasospasm was defined as symptomatic arterial narrowing requiring active intervention (e.g., intra-arterial vasodilators or balloon angioplasty) during hospitalization. Radiographic vasospasm without corresponding clinical symptoms was not considered relevant to outcome assessment. Ischemic infarcts were defined as new CT hypodensities developing after admission and attributable to surgery, vasospasm, or thromboembolic events. Postoperative seizures included any episode of status epilepticus or seizures necessitating medical treatment during hospitalization. Infections were recorded only in patients with positive cerebrospinal fluid cultures confirming bacterial or fungal growth.

### Outcome measures and data collection

Microsurgical clipping was performed using standard surgical approaches, and skull base approaches in some selected cases. Endovascular treatment included coiling alone, balloon-assisted or stent-assisted coiling, with the latter more commonly employed in later years as technology advanced.

### Aneurysm occlusion status

Aneurysm occlusion was assessed using DSA performed prior to hospital discharge. In the endovascular group, occlusions were classified with the Raymond-Roy classification [12, 13]. For comparative analysis, class 0 and class 1 were considered complete occlusion, while class 2 and 3 were considered incomplete. In the microsurgical group, results were categorized as complete or incomplete based on angiographic evaluation. In both treatment groups, occlusion completeness was determined using DSA whenever feasible. In cases where DSA could not be done, high-resolution CTA was used. Any visible neck remnant, regardless of size, was classified as incomplete occlusion.

### Retreatment

Retreatment was defined as subsequent interventions performed for a recurrent or residual AComA aneurysm that demonstrated morphological progression on follow-up imaging, evidence of rebleeding, or was judged by the treating team to carry a substantial rupture risk, including repeat coiling, stent-assisted coiling, or microsurgical clipping. Stable, small remnants without growth were managed conservatively with serial imaging. In the endovascular group, retreatment was analyzed in two ways: first, by including all secondary procedures regardless of intent; and second, by excluding cases managed with a pre-planned two-stage approach, for example by initial coiling followed by elective stent-assisted coiling within the first year. To ensure clarity in outcome classification, this strategy differentiated unplanned retreatment procedures from intentionally staged endovascular treatments defined at the initial intervention [14, 15]. Long-term treatment durability was assessed by comparing retreatment rates between the microsurgical and endovascular groups.

### Functional neurologic outcomes

Neurological recovery was assessed using the Glasgow Outcome Scale (GOS) at discharge, with good outcomes defined as scores of 4 or 5 and poor as 1 to 3. The modified Rankin Scale (mRS) was used to evaluate long-term functional status at 1-year and at the last follow-up, with good outcomes defined as scores of 0–2 and poor as 3–6. Outcome assessments were conducted by investigators who were not involved in any of these patient’s care.

### Statistical analyses

Statistical analyses were performed using IBM SPSS Statistics version 30.0 (IBM Corp., Armonk, NY). Continuous variables were presented as means ± standard deviations, and categorical variables summarized using frequencies and percentages.

The independent samples t-test was used for two-group comparisons of continuous variables based on distribution normality (assessed via the Kolmogorov-Smirnov test). The chi-square test or Fisher’s exact test were used for categorical variables, as appropriate.

Associations between aneurysm projection type and discharge outcome (good vs. poor) were assessed using the chi-square test of independence. When the overall test yielded statistical significance, post-hoc analysis of standardized residuals was conducted to identify specific group differences. A z-value greater than ± 1.96 was considered statistically significant.

Aneurysm sizes were compared between ruptured and unruptured groups using independent samples t-test and two-way analysis of variance (ANOVA). Projection type and rupture status were fixed factors, and aneurysm size was the dependent variable. Estimated marginal means and Bonferroni-corrected pairwise comparisons were used to evaluate subgroup differences.

Multivariate logistic regression analysis was used to identify independent predictors of poor outcome at discharge in the ruptured group. Variables with *p* < 0.10 in univariate analysis were entered into the final model. Model performance was evaluated using Nagelkerke R², the Hosmer–Lemeshow test, and classification accuracy.

Unless otherwise specified, statistical significance was set at *p* < 0.05 for all analyses.

## Results

### Patient demographics and treatment modality

The study included 313 patients with AComA aneurysms of which 166 (53.0%) were treated with microsurgical clipping and 147 (47.0%) with endovascular intervention. Among the microsurgical group, 99 (59.6%) patients had ruptured aneurysms, while in the endovascular group, 112 (76.2%) patients presented with rupture. Significant differences were observed in sex distribution (*p* = 0.021), rupture status (*p* = 0.002), and antithrombotic therapy (*p* = 0.002). In contrast, no statistically significant differences were found in age or vascular comorbidities between treatment groups (Table 1). Notably, the prevalence of diabetes mellitus (DM) was significantly higher in the unruptured group (27.5%) than in the ruptured group (8.1%) (χ²(1, *N* = 313) = 21.01, *p* < 0.001).

**Table 1:** Demographic and clinical characteristics of the study population by treatment modality

	Microsurgery (*n* = 166)	Endovascular (*n* = 147)	*p*-value	Test statistics
Age, years (mean ± SD)	57.02 ± 11.49	57.88 ± 11.98	0.518^t^	− 0.647
*Sex, n (%)*				
Male	86 (51.8%)	57 (38.8%)	0.021* ^x^	5.336
Female	80 (48.2%)	90 (61.2%)		
Hypertension, n (%)	83 (50%)	73 (49.7%)	0.952 ^x^	0.004
Diabetes mellitus, n (%)	27 (16.3%)	18 (12.2%)	0.312 ^x^	1.024
Smoking, n (%)	79 (47.6%)	81 (55.1%)	0.185 ^x^	1.760
Antithrombotic therapy use, n (%)	23 (13.9%)	41 (27.9%)	0.002* ^x^	9.442
*Aneurysm rupture status, n (%)*				
Ruptured	99 (59.6%)	112 (76.2%)	0.002* ^x^	9.723
Unruptured	67 (40.4%)	35 (23.8%)		

x: Chi square test, t: independent sample T test, *: *p* < 0.05

### Clinical presentation and perioperative complications

Among patients with ruptured AComA aneurysms, initial clinical status at admission did not differ significantly between treatment groups. Hunt-Hess grade II was the most frequently observed admission grade overall, with no significant differences found in Hunt-Hess or modified Fisher grades between the microsurgical and endovascular groups. Most patients had a Glasgow Coma Scale (GCS) score between 13 and 15 at presentation, with no significant difference between groups (*p* = 0.175). Radiological findings, including intracerebral hematoma (*p* = 0.604), midline shift (*p* = 0.256), and acute hydrocephalus (*p* = 0.245) were similar between groups. External ventricular drain (EVD) placement was significantly more common in the microsurgical group (*p* < 0.001), (Table 2). A statistically significant negative correlation was found between hospital stay and EVD status (1 = EVD inserted, 2 = no EVD) (*r* = − 0.308, *p* < 0.001). This indicates that patients without EVD tended to have shorter hospital stays, whereas longer stays were more common among those with EVD.

**Table 2:** Clinical presentation, complications, and early postoperative outcomes in ruptured AComA aneurysms by treatment modality

	Microsurgery (*n* = 99)	Endovascular (*n* = 112)	p-value	Test statistics
*Admission GCS, n (%)*				
15	43 (43.4%)	62 (55.4%)	0.175 ^x^	4.954
13–14	28 (28.3%)	29 (25.9%)		
9–12	11 (11.1%)	5 (4.5%)		
≤ 8	17 (17.2)	16 (14.3%)		
*Hunt-Hess grade, n (%)*				
Grade I	7 (7.1%)	6 (5.4%)	0.896 ^x^	1.090
Grade II	46 (46.5%)	57 (50.9%)		
Grade III	22 (22.2%)	27 (24.1%)		
Grade IV	17 (17.2%)	15 (13.4%)		
Grade V	7 (7.1%)	7 (6.3%)		
*Modified Fisher grade, n (%)*				
Grade I - II	15 (15.2%)	30 (26.8%)	0.065 ^x^	5.474
Grade III	61 (61.6%)	53 (47.3%)		
Grade IV	23 (23.2%)	29 (25.9%)		
Midline shift, n (%)	3 (3%)	1 (0.9%)	0.256 ^x^	1.291
Intracerebral hematoma, n (%)	9 (9.1%)	8 (7.1%)	0.604 ^x^	0.269
Acute hydrocephalus, n (%)	30 (30.3%)	26 (23.2%)	0.245 ^x^	1.354
EVD placement, n (%)	86 (86.9%)	62 (55.4%)	< 0.001** ^x^	24.916
Decompressive craniectomy, n (%)	11 (11.1%)	3 (2.7%)	0.014* ^x^	6.032
*SAH - Related Complications*				
Shunt-dependent hydrocephalus, n (%)	37 (37.4%)	39 (34.8%)	0.700 ^x^	0.149
Vasospasm, n (%)	44 (44.4%)	36 (32.1%)	0.066 ^x^	3.378
Any ischemic infarcts, n (%)	5 (5.1%)	8 (7.1%)	0.528 ^x^	0.398
Postoperative seizure, n (%)	24 (24.2%)	15 (13.4%)	0.043* ^x^	4.106
Rebleeding, n (%)	0	4 (3.6%)	0.058 ^x^	3.604
*Other complications*				
Infection, n (%)	5 (5.1%)	2 (1.8%)	0.186 ^x^	1.746
Early mortality (≤ 30 days), n (%)	7 (7.1%)	13 (11.6%)	0.262 ^x^	1.260
No clinical complications, n (%)	34 (34.3%)	48 (42.9%)	0.205 ^x^	1.603
Length of hospital stay, days (mean ± SD)	22.58 ± 11.38	18.46 ± 9.01	0.004*^t^	2.922

*EVD* External ventricular drainage, *SAH* Subarachnoid hemorrhage. x: Chi square test, t: independent sample T test, *: *p* < 0.05

Most complication types did not differ significantly between treatment groups; however, postoperative seizures (*p* = 0.043), and decompressive craniectomy (*p* = 0.014) were more common in the MC. Other complications, including vasospasm, new ischemia, early mortality, and postoperative infections, showed no statistically significant differences between treatment groups (p *>* 0.05). The mean length of hospital stay was significantly longer in the microsurgical group (22.6 ± 11.4 vs. 18.5 ± 9.0 days, *p* = 0.004) (Table 2). Early complications in the unruptured cohort were low in frequency and are summarized in Supplementary Table 1.

### Aneurysm size and projection characteristics

**Fig. 1:**
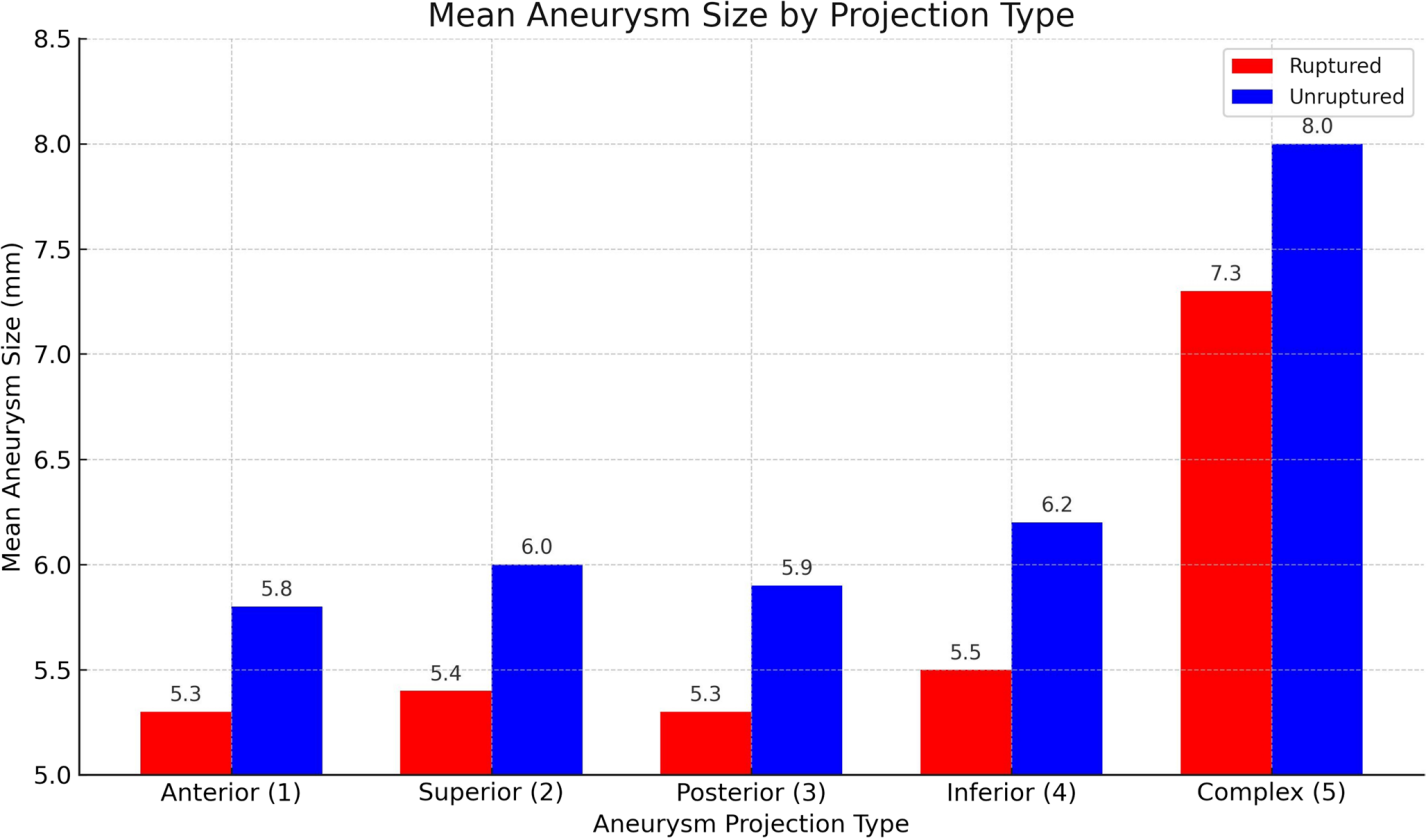
Comparison of mean aneurysm size (mm) across five projection types, stratified by rupture status (ruptured vs. unruptured). Complex projections were associated with significantly larger aneurysm sizes compared to anterior, superior, and posterior types (*p* < 0.05). Statistical analysis was performed using two-way ANOVA

The mean size of AComA aneurysms was significantly larger in the unruptured group (6.80 ± 3.81 mm) compared to the ruptured group (5.82 ± 3.30 mm, *p* = 0.020), (Table 3). Among the 313 AComA aneurysms, the most common aneurysm projections were superior (29.1%) and posterior (26.2%), followed by anterior (20.4%), complex (17.6%), and inferior (6.7%). Two-way ANOVA demonstrated that aneurysm size was significantly associated with both rupture status (*p* = 0.042) and projection type (*p* = 0.008), indicating that aneurysms differed in size depending on rupture presentation and anatomical orientation. No significant association was found between rupture status and projection type (*p* = 0.500), suggesting that projection-related size differences were consistent across groups. The mean aneurysm size was larger in the microsurgical group compared with the endovascular group (6.41 ± 4.02 mm vs. 5.84 ± 2.79 mm), but this difference was not statistically significant (t(311) = 1.42, *p* = 0.157). In terms of complex projection, the overall distribution across projection types was broadly similar between groups; however, complex projections (Type 5) were more frequent in the microsurgical group (22.9%, 38/166) than in the endovascular group (11.6%, 17/147). Post hoc Bonferroni tests demonstrated that aneurysms with complex projections (Type 5) were significantly larger than those with anterior (Type 1, *p* = 0.007), superior (Type 2, *p* = 0.015), and posterior (Type 3, *p* = 0.002) orientations (Fig. 1). MC reconstruction technique for a complex AComA aneurysm is demonstrated in Video 1.

**Table 3:** Comparison of mean aneurysm size and size categories between ruptured and unruptured aneurysms

	Ruptured (*n* = 211)	Unruptured (*n* = 102)	*p*-value	Test statistics
Aneurysm size (mm, mean ± SD)	5.82 ± 3.3	6.80 ± 3.81	0.02* ^t^	− 2.343
*Size category, n (%)*				
Small (< 5.0 mm)	88 (41.7%)	31 (30.4%)	0.036* ^x^	8.527
Medium (5.0–9.9)	102 (43.3%)	50 (49%)		
Large (10.0–24.9)	19 (9%)	20 (19.6%)		
Giant (≥ 25.0)	2 (0.9%)	1 (1%)		
*Aneurysm projection, n (%)*				
Anterior	40 (19%)	24 (23.5%)	< 0.001** ^x^	32.193
Superior	77 (36.5%)	14 (13.7%)		
Posterior	58 (27.5%)	24 (23.5%)		
Inferior	14 (6.6%)	7 (6.9%)		
Complex	22 (10.4%)	33 (32.4%)		

x: Chi square test, t: independent sample T test, *: *p* < 0.05

### Angiographic outcomes and retreatment rates

Complete aneurysm occlusion was achieved in 163 of 166 patients (98.2%) treated with MC, compared to 56 of 147 patients (38.1%) treated with ET (*p* < 0.001). Regarding the initial endovascular technique, ruptured aneurysms were managed predominantly with primary coiling (108/112; 96.4%), whereas stent-assisted coiling was performed in 4 cases (3.6%). In the unruptured cohort, 18 of 35 patients (51.4%) underwent coiling alone, and 17 (48.6%) underwent stent-assisted coiling. Retreatment was performed in 30 patients (20.4%) in the endovascular group, while no retreatment was required for the microsurgical group (*p* < 0.001, Fisher’s exact test). Among the 30 retreated endovascular cases, 7 patients underwent planned second-stage procedures (all stent-assisted coiling within the first year) based on aneurysm morphology. After excluding the seven planned second-stage procedures, the retreatment rate in the endovascular group was 15.6% (23/147), which remained significantly higher than in the microsurgical group, where no retreatments occurred (*p* < 0.001) (Table 4). To account for potential changes in endovascular practice over time, a subgroup analysis was performed including only patients treated between 2015 and 2024 (*n* = 190; 88 MC, 102 ET). In this recent cohort, complete occlusion was achieved in 100% (88/88) of patients in the microsurgical group and 41.2% (42/102) of the endovascular group (χ²(1) = 75.656, *p* < 0.001). Retreatment for recurrence or regrowth was required in 0% (0/88) of microsurgical patients compared with 13.7% (14/102) of endovascular patients (χ²(1) = 13.039, *p* < 0.001).

**Table 4:** Angiographic and clinical outcome comparison between microsurgical and endovascular treatment groups

	Microsurgical (*n* = 166)	Endovascular (*n* = 147)	*p*-value	Test statistics
Complete occlusion	163 (98.2%)	56 (38.1%)	< 0.001** ^x^	134.002
Retreatment	0	30 (20.4%)	< 0.001** ^f^	37.469
Retreatment (excluding planned second-stage procedures)	0	23 (15.6%)	< 0.001** ^f^	28.033
Follow-up period (months)	56.56 ± 50.65	59.40 ± 44.71	0.311	0.818

Pearson Chi-square test was used since the assumption of minimum expected cell frequency (> 5) was met for all cells. *f*: Due to small expected cell counts the Fisher’s exact test was used instead of Pearson’s chi-square test to assess group differences, **: *p* < 0.001

Of the three patients in the microsurgical group with incomplete occlusion, one died within 30 days due to SAH–related complications; this patient had a poor clinical grade at admission and multiple comorbidities. The remaining two patients had no recurrence or hemorrhage during the available follow-ups (19 and 63 months). Among the 30 endovascular patients who underwent retreatment, 9 had persistent residual filling and 21 experienced aneurysm recurrence. Additional patients in the endovascular group with aneurysm remnants or radiographic recurrence were managed conservatively and followed both clinically and radiographically. These decisions were based on factors that included patient preference, procedural risk, and minimal regrowth over time.

Within the endovascular group, the retreatment rate was higher among patients with ruptured aneurysms (23.2%, *n* = 26) compared to those without rupture (11.4%, *n* = 4), although this difference did not reach statistical significance (χ²(1) = 2.280, *p* = 0.131). Among those who underwent retreatment, five patients ultimately required conversion to microsurgical clipping, including four with ruptured aneurysms and one without. Video 2 illustrates a subsequent MC following ET, performed 3 years after the initial treatment.

Hemorrhage following initial treatment occurred in four patients in the endovascular group (2.7%). Three of these events occurred within 72 h, including one in a patient with an unruptured aneurysm. The fourth case was a delayed hemorrhage in a patient with a previously ruptured aneurysm. No such events occurred in the microsurgical group (*p* = 0.048).

### Functional neurologic outcomes

**Fig. 2:**
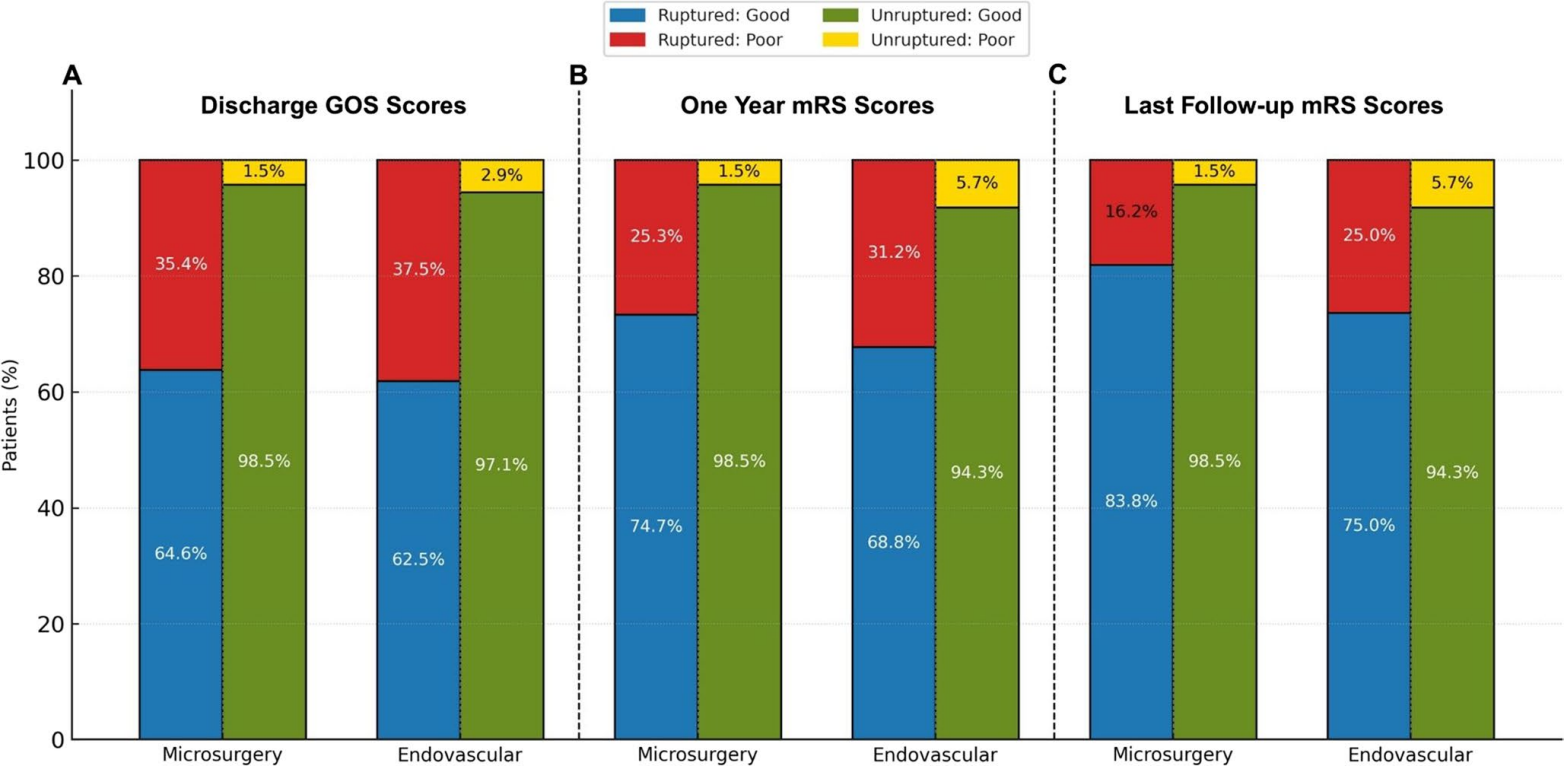
Stacked bars compare microsurgical and endovascular cohorts at (**A**) discharge, (**B**) one year, and (**C**) last follow-up. Bars are stratified by rupture status: the left half of each bar represents ruptured aneurysms, and the right half represents unruptured aneurysms. Favorable outcome was defined as GOS 4–5 at discharge and mRS 0–2 at 1 year and last follow-up (poor = GOS 1–3 or mRS 3–6)

Functional outcomes following treatment of AComA aneurysms were evaluated separately for ruptured and unruptured subgroups. In the ruptured aneurysm group, good outcomes at discharge (GOS 4–5) were observed in 64.6% of microsurgically treated patients and 62.5% of those treated endovascularly (*p* = 0.747). At the one-year follow-up, favorable outcomes (mRS 0–2) were recorded in 74.7% of the microsurgical group and 68.8% of the endovascular group (*p* = 0.335). At the last follow-up, good recovery was achieved in 83.8% of microsurgical group patients and 75.0% of endovascular patients (*p* = 0.115) (Fig. 2). In the unruptured aneurysm group, good functional outcomes at discharge (GOS 4–5) were achieved in 98.5% of microsurgical and 97.1% of endovascular cases (*p* = 0.637). At one-year follow-up, favorable outcomes (mRS 0–2) were observed in 98.5% and 94.3%, respectively (*p* = 0.231). These rates remained unchanged at last follow-up (98.5% vs. 94.3%, *p* = 0.231).

### Predictors of poor outcome

A significant association was found between aneurysm projection type and discharge outcome (χ²(4) = 15.67, *p* = 0.003). Post hoc analysis of standardized residuals indicated that patients with complex aneurysm projections were significantly more likely to have poor outcomes than expected (z = + 2.8), while fewer than expected had good outcomes (z = − 2.1).

Multivariate logistic regression analysis indicated that increasing age was significantly associated with poor clinical outcomes at discharge for the ruptured aneurysms group (OR = 1.088, *p* < 0.001). Among clinical variables, postoperative cerebral infarction (OR = 0.038, *p* = 0.005) and vasospasm requiring active intervention (OR = 0.200, *p* < 0.001) emerged as independent predictors of unfavorable outcomes. Aneurysm size demonstrated a non-significant trend toward association with outcome (OR = 1.106, *p* = 0.059). The overall model showed strong predictive capacity (Nagelkerke R² = 0.541) and good calibration (Hosmer–Lemeshow *p* = 0.830), with a correct classification rate of 79.6%.

## Discussion

### Summary of key findings

AComA aneurysms are among the most complex lesions in cerebrovascular surgery, given their deep midline location, frequent anatomical variations, and critical perforator involvement. These factors contribute to the persistent dilemma in selecting between MC and ET, as each modality presents distinct advantages, challenges, and risk profiles [16, 17].

This retrospective comparative study evaluates the long-term outcomes of MC and ET in a large group of ruptured and unruptured AComA aneurysms. The most striking finding was the significantly greater rate of durable occlusion achieved with MC (98.2%). Moreover, while no retreatments were required in the microsurgical group, a notable subset of endovascularly treated patients required additional procedures during follow-up. These results are consistent with prior studies demonstrating better obliteration rates and a lower incidence of retreatment with MC [7, 18–20].

### Durability of treatment modalities

ET is widely preferred in many centers due to its minimally invasive nature and lower periprocedural morbidity, as demonstrated in major trials and institutional series [21–23]. However, its long-term treatment durability remains a major limitation. Herein we found no cases of retreatment or postoperative hemorrhage in the microsurgical group. In contrast, 30 patients (20.4%) in the endovascular group required retreatment, 7 of which were planned second-stage procedures, and notably, post-treatment hemorrhage occurred exclusively in endovascularly treated patients. While these outcomes raise durability concerns, the higher retreatment rate—especially in ruptured cases—may reflect a strategy focused on early dome protection, with definitive treatment deferred. These angiographic durability differences remained evident even when limiting the analysis to the most recent decade (2015–2024), a period marked by greater adoption of stent-assisted coiling and other endovascular technical advances. In this subgroup, microsurgical clipping maintained a 100% complete occlusion rate with no retreatments, whereas endovascular treatment achieved complete occlusion in only 41.2% of cases and required retreatment in 13.7%. These findings suggest that the superior long-term durability of clipping over coiling in our cohort is not merely attributable to earlier-era limitations of endovascular technology. Overall, the higher rates of incomplete occlusion and retreatment observed in endovascular group patients suggest that ET may need more frequent long-term radiological surveillance to detect recurrence or regrowth. These findings should also be interpreted in light of the substantial baseline differences—particularly rupture presentation—between the treatment cohorts, which inherently limit the strength of direct cross-modality comparisons.

### Complications and clinical outcomes

While some studies have associated MC with higher rates of postoperative ischemia, particularly for complex aneurysms, others have reported comparable outcomes for ET in experienced centers [24, 25]. In our cohort, rates of ischemia did not differ significantly between treatment groups. MC did have a higher incidence of postoperative seizures; however, overall complication profiles remained comparable. These findings suggest that MC, when performed in high-volume centers, is not associated with increased morbidity and ensures long-term aneurysm control. The higher rate of decompressive craniectomy in the microsurgical group may relate to the greater proportion of patients presenting with poor-grade SAH or mass effect, although these differences did not reach statistical significance and should be interpreted as a clinical trend rather than a confirmed association.

### Aneurysm morphology and rupture characteristics

The mean size of ruptured AComA aneurysms in our study population was significantly smaller than the mean size of unruptured aneurysms. This supports the notion that small aneurysm size does not confer protection in this anatomical location. Although early large-scale studies such as the International Study of Unruptured Intracranial Aneurysms (ISUIA) and the Unruptured Cerebral Aneurysm Study of Japan (UCAS Japan) suggested that aneurysms < 7 mm in the anterior circulation may be safely observed [26, 27], subsequent reports have challenged this assumption regarding AComA aneurysms [28, 29], showing that morphological features such as higher aspect ratios and smaller neck diameters contribute to rupture risk [30]. A meta-analysis demonstrated that 67% of ruptured AComA aneurysms were smaller than 7 mm [31]. In the present study, the mean ruptured aneurysm diameter was 5.82 mm, further supporting the notion that size alone is insufficient for reliable risk stratification. Clinical decision-making should consider the morphological complexity and associated hemodynamic stress factors [32, 33]. In our study population, aneurysms with complex projections were also significantly larger in cases of rupture.

DM was significantly more prevalent in unruptured aneurysms. This inverse association aligns with previous reports. For instance, Zhong et al. (2023) reported a lower prevalence of DM among patients presenting with aneurysmal SAH [34], and a meta-analysis of 18 studies also showed a reduced relative risk of aneurysmal SAH in diabetic individuals [35]. Potential mechanisms remain speculative, but some studies suggest that DM may contribute to aneurysm wall stabilization through atherosclerotic changes, particularly in elderly patients [36, 37]. These observations collectively suggest that DM may exert a protective effect against rupture, but further studies are needed to confirm this association.

### Functional outcomes and comparison with existing literature

Among patients with ruptured AComA aneurysms, we observed favorable functional outcomes at one year in 74.7% of those treated microsurgically and 68.8% of those treated endovascularly. Our microsurgical outcomes were slightly better and endovascular outcomes slightly worse compared with those reported in ISAT [38]. These findings may reflect differences in institutional protocols, surgical expertise, patient selection criteria, or baseline neurological status.

The present study contributes to existing evidence which suggests the early benefits of ET may not translate into long-term advantages. Recent meta-analyses and large-scale studies have shown that while ET offers reduced procedural morbidity in select cases, MC provides more durable aneurysm obliteration without compromising long-term functional independence [24, 39]. Moreover, in high-volume centers with established surgical expertise, the rate of favorable outcomes following clipping may equal or even exceed those of coiling [40]. These observations highlight the importance of considering aneurysm morphology, rupture status, available resources, and the experience of the treating team in achieving optimal patient outcomes. Both microsurgical clipping and endovascular treatment are operator-dependent procedures, and differences in institutional expertise may influence clinical outcomes and long-term durability.

### Predictors of outcome

In the present analysis, postoperative cerebral infarction and vasospasm requiring active intervention emerged as the strongest independent predictors of poor clinical outcome at discharge among patients with ruptured AComA aneurysms. These findings align with prior studies identifying secondary ischemia and vasospasm as key contributors to unfavorable early outcomes following aneurysmal SAH [41–45]. Older age was also associated with poor outcomes, consistent with evidence that elderly patients have reduced neurological resilience and higher risk of post-hemorrhagic complications [46–48]. Altogether, these findings underscore the importance of targeted strategies to minimize ischemic injury and optimize vasospasm management, thereby improving recovery and prognosis.

To contextualize our findings, we summarized and compared data from major contemporary studies evaluating MC and ET of AcomA aneurysms (Table 5). Across most series, MC demonstrated higher rates of complete occlusion and lower recurrence or retreatment rates, whereas ET showed lower procedural morbidity and comparable long-term functional outcomes. These consistent trends—also observed in our cohort—emphasize that although ET remains less invasive, MC continues to provide the most durable and definitive aneurysm exclusion.

**Table 5:** Summary of comparative studies comparing microsurgical clipping and endovascular treatment outcomes in AComA aneurysms

Study (Year)	Design/Population (*N*)	Treatment modality (*N*, MC/ET)	Complete occlusion (%)	Retreatment (%)	Post-treatment rebleeding (%)	Good functional outcome	Follow-up (months)	Notes
Present series 2025	Single-center retrospective; 211 Ruptured + 102 Unruptured (*n* = 313)	MC 166/ET 147	MC 98.2%/ET 38.1%	MC none/ET 15.6% (excluding staged)	MC none/ET 2.7%	Ruptured: MC 83.8%/ET 75.0%; Unruptured: MC 98.5%/ET 94.3% (at last FU)	MC 56.6 ± 50.7/ET 59.4 ± 44.7	Durable occlusion significantly higher with MC; functional outcomes comparable between groups.
Proust et al., 2003 [49]	Prospective; predominantly ruptured; 214 ruptured/9 unruptured (*n* = 223)	MC 186/ET 37	MC 91.9%/ET 78.4%	NR	MC 1.1%/ET 2.7%	Ruptured: MC 61.4% (early period)/71.8% (late period)/ET 54.1%; Unruptured: MC 100% (MC only) (GOS = 5)	6–12	Higher occlusion with MC; ET had more remnants, lower morbidity, and slightly worse outcomes.
Proust et al., 2009 [50]	Prospective; ruptured only (*n* = 50)	MC 36/ET 14	NR (residue: MC 13.9%, ET 35.7%)	NR	MC 0%/ET 7.1%	MC 88.9%/ET 92.9%	14	No significant differences in global functional outcome.
Spetzler et al. 2015 [22]	Prospective, randomized; ruptured only (*n* = 408)	MC 209/ET 199	MC 96%/ET 48%	MC 4.6%/ET 16.4%	None in both	MC 59%/ET 65%	72	Durable occlusion higher with MC; similar functional outcomes.
Steklacova et al., 2017 [31]	Single-center retrospective; 304 ruptured, 94 unruptured (*n* = 398)	MC 79/ET 319	NR	MC 3.8%/ET 9.2%	NR	Ruptured: MC 74%/ET 70%; Unruptured: MC 100%/ET 98.5%	NR	MC achieved higher durability; ET associated with higher recurrence.
Zhao et al., 2019 [51]	Single-center retrospective; ruptured only, very small (≤ 3 mm) (*n* = 111)	MC 65/ET 46	NR	None	No significant difference	MC 84.5%/ET 89.2% (GOS 4–5, at discharge)	32.4 ± 22.2	Comparable outcomes between MC and ET for very small ruptured aneurysms.
Moon et al., 2020 [18]	Retrospective; unruptured only (*n* = 70)	MC 33/ET 37	MC 90%/ET 78.4%	MC 10%/ET 5.6%	NR	MC 100%/ET 97.3%	15	Unruptured only; excellent and comparable outcomes.
Ki et al., 2020 [52]	Single-center retrospective; 48 ruptured, 166 unruptured (*n* = 214)	MC 105/ET 109	Overall occlusion ~ 90% (no subgroup data)	NR	NR	NR	36.9 ± 18.4	Recurrence observed in 13% overall; outcomes comparable.
Harris et al., 2021 [53]	Single-center retrospective; ruptured only (*n* = 137)	MC 19/ET 113	MC 94.7%/ET 80.5%	MC none/ET 10.6%	MC 0%/ET 5.3%	MC 57.9%/ET 78.4% (at discharge)	33.2	Increased recurrence risk with ET at 1 year.
Haeren et al., 2022 [54]	Single-center retrospective; unruptured only (*n* = 128)	MC 81/ET 47	MC 96%/ET 89%	MC 1%/ET 4%	MC 0%/ET 2%	MC 86%/ET 97% (mRS 0–1)	6 (ET only)	No overall difference in major complications; functional outcomes comparable.
Lee & Park, 2022 [55]	Single-center retrospective; ruptured only (*n* = 213)	MC 94/ET 119	NR	NR	MC 3.2%/ET 4.2%	MC 57.4%/ET 73.1%	3 (primary outcome)	Coiling independently associated with favorable outcome.
Yang et al., 2024 [56]	Multicenter retrospective observational; ruptured only (*n* = 893)	MC 346/ET 549 (Propensity score matching: 275 pairs)	NR	MC 1.8%/ET 1.8%	NR	MC 75.6%/ET 85.5% (2 years FU)	24	Total procedural complications lower with ET (14.2% vs. 21.1%); 2-year mortality lower with ET.
Swiatek et al., 2025 [24]	Single-center retrospective; 111 ruptured, 29 unruptured (*n* = 140)	MC 24/ET 116	MC > ET (*p* = 0.007)	Revision for incomplete occlusion: MC 33%/ET 13% (subset only)	NR	No significant difference at discharge and follow-up	Institutional (duration not specified)	MC associated with higher ischemic complications; functional outcomes comparable.

*MC* microsurgical clipping, *ET* endovascular treatment, *FU* follow-up, *NR* not reported. Good functional outcome was generally defined as mRS 0–2 or GOS 4–5; deviations from this definition and differences in follow-up timepoints are specified in the Notes column

## Limitations

This study has several limitations. First, its retrospective design introduces risks of selection and information bias. Second, although long-term outcomes were reported, variability in follow-up duration and imaging protocols may have influenced the detection of aneurysm recurrence or the need for retreatment. DSA was the primary follow-up modality and was obtained whenever feasible, reflecting our center’s preference for high-resolution angiographic assessment. CTA was used only when DSA could not be performed. Although CTA is less sensitive for detecting small neck remnants, its limited and selective use makes any underdetection unlikely to have influenced the comparative conclusions. Additionally, the vascular access route (transfemoral vs. transradial) was not documented in the dataset, preventing assessment of access-related complication differences. Third, while functional outcomes were evaluated using standardized scales, subtle neurocognitive deficits may have gone undetected. Fourth, the nearly two-decade study period (2006–2024) may not fully reflect current endovascular techniques or perioperative advances. However, a subgroup analysis limited to the most recent decade (2015–2024) demonstrated that the observed differences in angiographic durability between modalities persisted despite greater adoption of stent-assisted coiling and other technical improvements. Additionally, differences in rupture presentation and aneurysm morphology between the treatment groups—particularly the higher frequency of complex projections in the microsurgical cohort—may introduce confounding by indication and limit the strength of direct comparisons. Furthermore, some subgroup analyses—such as comparisons between ruptured and unruptured aneurysms or between simple and complex morphology—contained relatively small patient numbers, which may have reduced statistical power. Lastly, generalizability may be limited, particularly in centers with differing degrees of microsurgical and/or endovascular expertise.

## Conclusions

This study highlights the continued relevance of MC for the management of AComA aneurysms, particularly in terms of long-term durability and occlusion rates. Although endovascular coiling remains the less invasive option, its higher retreatment and lower occlusion rates emphasize the need for careful patient selection and close follow-up. Our findings also reinforce the notion that small aneurysm size does not provide protection in the AComA region, underscoring the importance of incorporating morphological and hemodynamic factors into the assessment of rupture risk and treatment decisions, while recognizing that differences in rupture presentation between the treatment groups should be considered when interpreting these findings.

## Supplementary Information

Below is the link to the electronic supplementary material.

Video 1: A 53-year-old female with an incidentally discovered, unruptured, complex multilobed anterior communicating artery aneurysm underwent microsurgical clipping via a right pterional craniotomy 

Video 2: A 44-year-old male with prior subarachnoid hemorrhage and coil-treated anterior communicating artery aneurysm presented with recurrence, and subsequently underwent microsurgical clipping via a right pterional approach

Supplementary Material 3

## Data Availability

No datasets were generated or analysed during the current study.
