# Supplementary material for: Comparative outcomes of microsurgical and endovascular treatment for ruptured and unruptured anterior communicating artery aneurysms
Source: Neurosurg Rev. 2026 Jan 14;49(1):123. doi: 10.1007/s10143-025-04035-6 (PMC12799692; doi:10.1007/s10143-025-04035-6)
Supplement: Supplementary file 3 — Supplementary Material 3 [file 10143_2025_4035_MOESM3_ESM.docx]

**Supplementary Table 1** Periprocedural complications and length of hospital stay in unruptured

AComA aneurysms by treatment modality

|  | **Microsurgery (n = 67)** | **Endovascular (n = 35)** | **P-value** | **Test statistics** |
| --- | --- | --- | --- | --- |
| **Shunt-dependent hydrocephalus, n (%)** | 1 (1.5%) | 0 (0%) | 1.000 ^x^ | 0.528 |
| **Any ischemic infarcts, n (%)** | 1 (1.5%) | 2 (5.7%) | 0.231 ^x^ | 3.905 |
| **Intraprocedural rupture, n (%)** | 0 (0%) | 2 (5.7%) | 0.116 ^x^ | 3.905 |
| **Infection, n (%)** | 0 (0%) | 0 (0%) | — | — |
| **Postoperative seizure, n (%)** | 3 (4.5%) | 1 (2.9%) | 0.689 ^x^ | 0.160 |
| **Length of hospital stay, days (mean ± SD)** | 6.69 ± 6.26 | 3.80 ± 4.44 | 0.017 ^t^ | 2.427 |

x: Chi square test, t: independent sample T test, Pearson Chi-square test was used since the assumption of minimum expected cell frequency (>5) was met for all cells. *f*: Due to small expected cell counts the Fisher's exact test was used instead of Pearson's chi-square test to assess group differences
